# Effectiveness of Uvulopalatoplasty and Nasal Surgery in OSAS Patients

**DOI:** 10.22038/IJORL.2023.60817.3098

**Published:** 2023-07

**Authors:** Gegham Khandanyan, Anna Shukrian, Artur Potosyan, Parounak Zelveian, Artur Shukuryan

**Affiliations:** 1 *Department of ENT, Yerevan State Medical University, “Erebouni” Medical Center, Yerevan, Armenia.*; 2 *Unimed Medical Centre, Yerevan- Armenia.*; 3 *Institute of Cardiology Named after Levon Hovhannisyan, Yerevan-Armenia.*

**Keywords:** OSAS, Snoring, Uvulopalatoplasty, Nasal surgery

## Abstract

**Introduction:**

This study aims to evaluate the effectiveness of simultaneous uvulopalatoplasty and nasal surgery in patients with moderate obstructive sleep apnoea syndrome.

**Materials and Methods:**

We studied 48 patients with obstructive sleep apnoea syndrome and nasal breathing disorders. The 1^st^ group of 20 patients underwent septoplasty and volumetric tissue reduction of inferior turbinates and the 2^nd^ group of 28 patients underwent septoplasty, volumetric tissue reduction of inferior turbinate, uvulopalatoplasty.

**Results:**

In the 1^st^ group, the pre-operative apnoea-hypopnea index median decreased from 22.1 ep/h to 14.9 ep/h after the surgery. Pre-operative median of nasal airflow volume grew from 167.0 cm^3^ to 609.5 cm^3^ post-operatively and the loudness of snoring decreased from 2.7±0.2 to 0.7±0.2. In the 2^nd^ group, the apnoea-hypopnea index median decreased from 20.4 ep/h to 5.3 ep./h. The pre-operative median of nasal airflow volume grew from 189.5 cm^3^ to 519.5 cm^3^ post-operatively and the loudness of snoring improved from 2.6±0.2 pre-op to 1.1±0.2.

**Conclusion:**

Patients with moderate obstructive sleep apnoea syndrome and nasal breathing disorders are advised to have nasal surgery combined with uvulopalatoplasty for a better outcome.

## Introduction

Sleep-related breathing disorders generally exhibit a range of clinical forms - from primary snoring to severe Obstructive Sleep Apnoea Syndrome (OSAS) (1). OSAS is one of the major problems of medicine (2). According to international epidemiological data, OSAS occurs in 2-4% of the population with male to female ratio of 2:1 (3).

Since nasal airways can be viewed as “gates” of the respiratory tract, nasal airway disorders play a major role in the etiopathogenesis of sleep-related breathing conditions (4,5).

 Many authors have demonstrated that the difficulty of nasal breathing can be the cause of developing snoring, general fatigue, daytime sleepiness and OSAS (6-8), while other authors have argued that the degree of nasal breathing difficulty cannot be explained solely by nasal obstruction and that difficult nasal breathing cannot be the cause of moderate or severe obstructive sleep apnea (9,10).

Thus, in the presence of the two above-mentioned approaches, determining the necessity of intranasal surgical intervention plays an important role in the evaluation of OSAS treatment effectiveness. The aim of this study is to evaluate the effectiveness of simultaneous uvulopalatoplasty (UPP) and nasal airway surgery in patients with moderate OSAS. 

## Materials and Methods

The study was conducted in Yerevan, Armenia at the Department of Otorhinolaryngology (ENT), “Erebouni” Medical Center and Sleep Laboratory of the Institute of Cardiology from 2008 to 2016. 

This study has been approved by the Ethics Committee (EC) of “Erebouni” MC (11.09.2008, protocol №3) in Yerevan, Armenia. The study sample included 48 symptomatic patients (30 men and 18 women) between the age range of 27 to 62. All study subjects had breathing difficulty, pronounced snoring (confirmed by a person sharing the bedroom), choking during sleeping, along with morning headaches and daytime fatigue and sleepiness. 

All patients underwent detailed ENT examination as well as rhino-fibroscopy. Thorough sleep history was taken. The diagnosis in all patients was realised through anterior rhinoscopy and nasal endoscopic examination.

Paranasal sinus disease was diagnosed based on signs, symptoms, history, and when required, X-ray or CT-imaging.

Patients who had (a) nasal breathing difficulty; (b) a thickened or hanging soft palate; and (c) apnoea-hypopnea (AH) syndrome varying between 15-25 episodes per hour were selected for the study. They all had complaints about breathing difficulty, snoring, frequently - fatigue and occasionally – headaches and daytime sleepiness. Only patients with difficult nasal breathing due to deviated nasal septum with or without external nasal deformities, as well as unilateral or bilateral hypertrophied inferior turbinates were included in the study.

Patients with (a) chronic inflammatory nasal and sinus pathologies; (b) hypertrophy of palatine tonsils and root of tongue potentially causing choking or apnoea during sleep; and (c) patients who have already undergone surgery to reduce the inferior turbinates or the soft palate and whose AH syndrome was more than 25 episodes per hour were excluded from the study. Participants were divided into 2 groups according to the surgical intervention performed. 

The first group included 20 patients who underwent surgery to restore nasal airway patency - nasal septal submucosal resection when needed and radiofrequency volumetric tissue reduction (RVTR) of inferior turbinates. The second group included 28 patients who underwent RVTR of inferior turbinate and middle portion of the soft palate, and radiofrequency (RF) uvulopalatoplasty (UPP).

Body mass index (BMI) readings, severity of obstructive sleep apnoea according to the apnoea-hypopnea index (AHI), nasal breathing, loudness of snoring, and severity of day time sleepiness according to the Epworth sleepiness scale (ESS) were recorded before and after surgery.

The palatine tonsils were of 1^st^ or 2^nd^ degree sizes according to the Friedman classification in all participants.

The roots of the tongue and soft palate were also either 1^st^ or 2^nd^ degrees (11).

Sleep related breathing disorders were recorded and evaluated by EMBLA N7000 (EMBLA System, Inc.) polysomnography system using Somnologica v. 4.0 (EMBLA System, Inc.) software. Characterization of sleep was made using Reichtschaffen and Kales international criteria for sleep/wake determination from the American Sleep Disorders Academy (ASDA) (12). The severity of sleep disorders was quantified by using AHI that counts the total number of apnoea and hypopnea episodes in an astronomical hour. Five or more episodes per hour are considered pathological. Nasal airflow was measured by active anterior rhinomanometry. 

Patients wore tightfitting face masks, and breathed through one nostril keeping the mouth closed. A sensor placed in the contralateral nostril recorded data from pre-nasal pressures via airflow and pressure transducers. The equipment (4Phase Rhino-Lab Germany) was connected to a personal computer. The trans- nasal airflow and pressure signals were amplified, digitised, and stored for statistical analysis. 

Nasal airflow was reported as the sum of recorded airflow through the right and left nostrils in millilitres per second at a pressure difference of 150 Pa across the nasal passage. Three airflow measurements were performed for each patient and the mean was recorded when reproducible values were achieved. 

Nasal obstruction and conductance were graded using a 1-5 grade scale (13) (Table 1). 

 As for snoring, a 1-5 grade scale was designed according to the loudness of the sound. It ranges from 0-4 degrees (Table 2).

**Table I:** Clinical classification of nasal obstruction and conductance

**Grade**	**Conductance**	**Flow at 150Pa (cm** ^3^ **/s)**
1	Very high	> 500
2	High	300 - 500
3	Moderate	180 - 300
4	Low	60 - 180
5	Very low	< 60

**Table 2:** Snoring VAS

0	No snoring
1	Soft snoring that does not interrupt the bed partner’s sleep
2	Loud snoring - enough to bother the partner
3	Very intense snoring annoying everyone nearby
4	The bed partner leaves room

Inferior turbinate reduction and UPP procedures were carried out using the Ellman Surgitron Dual-Frequency IEC-II machine.

After infiltrating the tissue with 1% Lidocaine mixed with 1:100000 Epinephrine, RF UPP was performed using a radiofrequency surgical knife. A triangular excision was made on the hypertrophied mucosa of the posterior arch of the soft palate on both sides of the uvula. 

The inferior portions of the posterior arch were also excised preserving the muscular tissue when the soft palate droop was significant. Radiofrequency volumetric tissue reduction (RVTR) of soft palate tissue along the lateral segments of uvula was performed using a unipolar electrode at the same time. Patients with a uvula exceeding 1.5 cm of length underwent further uvulectomy with the RF surgical knife during the RF UPP procedure.

The study participants were prescribed nasal decongestants and antibiotics (mainly from the Amoxicillin/ Clavulonate group), and pharyngeal gargling for 7-10 days postoperatively.

***Data collection and statistical analysis***

A non-parametric statistical test was used due to the small size of the sample (n_1_=20 and n_2_=28). The non-parametric Wilcoxon signed-rank test for independent samples was applied to compare the quantitative parameters of pre- and post-surgical data. 

The Kruskal-Wallis test for independent samples was applied for making comparisons within the sample group. As for classified (qualitative) parameters, comparison was made using non-parametric Pearson’s chi-squared test (*χ*^2^). The level of marginal significance of p<0.05 was used in statistical analyses to reject the null hypothesis. Data collection and analysis were performed using MS Excel 2010 and SPSS 22.0 (SPSS, Inc., Chicago, IL, USA), taking into account and coordinating all the data shortcomings.

## Results

*First group*

There were 20 subjects in the first group aged 27 to 61, (mean age of 43.9 and median age of 44); 14 male (70%) and 6 female (30%) participants. Table 3 presents the postoperative changes in the studied parameters for the first group. The BMI records of the first group did not show any significant difference post-operation vs pre-operation, while the remaining parameters improved after surgery (Table 3). 

**Table 3:** Pre and post-operative parameters of difficult nasal breathing in patients with moderate OSAS

**Variable**	**Pre-Op (n=20)**	**Post-Op(n=20)**	**p value**
BMI (kg/m^2^), median	31.1 (33.6)	31.1 (33.3)	> 0.15 *
Apnoea-hypopnea index (ep./hour), median	21.7 (22.1)	14.2 (14.9)	< 0.05*
subgroups of apnoea-hypopnea index, absolute number (percentage)			
0^0 ^(normal, < 5 ep./hour)	0 (0.0)	0 (0.0)	-
I^0^ (mild, 5-15 ep./hour)	0 (0.0)	10 (50.0)	< 0.05**
II^0 ^(moderate, 15-25 ep./hour)	20 (100.0)	10 (50.0)	< 0.05**
III^0 ^(severe, > 25.ep./hour)	0 (0.0)	0 (0.0)	-
Nasal breathing difficulty (cm^3^), median	163.3 (167.0)	609.9 (605.5)	< 0.05*
Subgroups of Nasal breathing difficulty, absolute number (percentage)			
0^0 ^(normal, > 500 cm^3^)	0 (0.0)	17 (85.0)	< 0.05**
I^0^ (mild, 300-500 cm^3^)	0 (0.0)	3 (15.0)	< 0.05**
II^0^ (moderate, 180-300 cm^3^)	6 (30.0)	0 (0.0)	< 0.05**
III^0 ^(severe, 60-180 cm^3^)	10 (50.0)	0 (0.0)	< 0.05**
IV^0 ^(very severe, <60 cm^3^)	4 (20.0)	0 (0.0)	< 0.05**
Snoring, absolute number (percentage)			
No snoring	0 (0.0)	11 (55.0)	< 0.05**
I^0 ^(mild)	1 (5.0)	5 (25.0)	< 0.05**
II^0^ (moderate)	8 (40.0)	3 (15.0)	< 0.05**
III^0^ (severe)	7 (35.0)	1 (5.0)	< 0.05**
IV^0^ (very severe)	4 (20.0)	0 (0.0)	< 0.05**
Epworth sleepiness scale, (median)	9.5 (10)	2.2 (2)	< 0.05*

*Note.* *Wilcoxon statistical test, **Pearson statistical test

*Particularly,*

**Fig 1:**
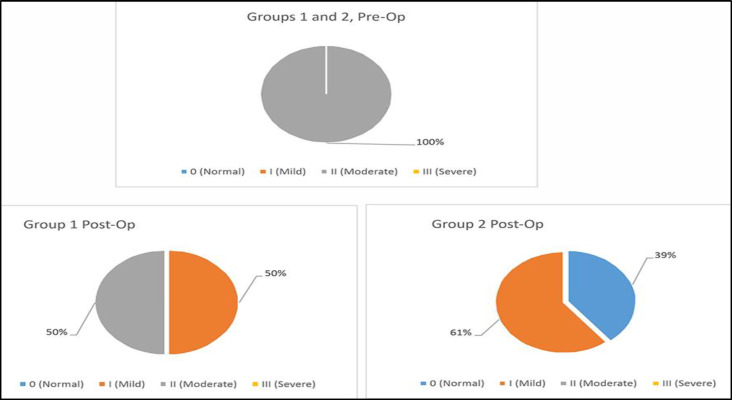
Comparative characteristics of Apnea-Hypopnea index in Groups 1 and 2 pre- and post-operation

**Fig 2:**
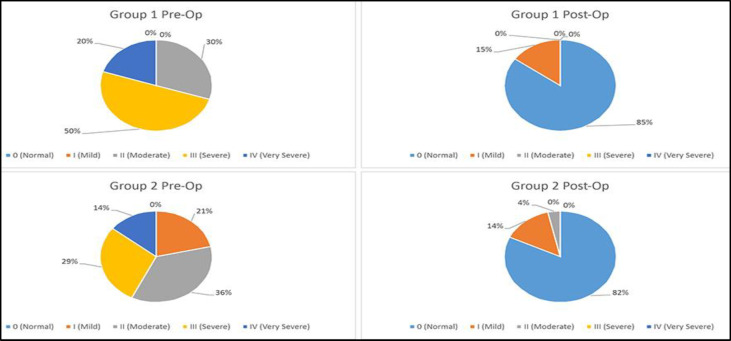
Comparative characteristics of nasal breathing Groups 1 and 2 pre- and post-operation interventions

**Figure 3:**
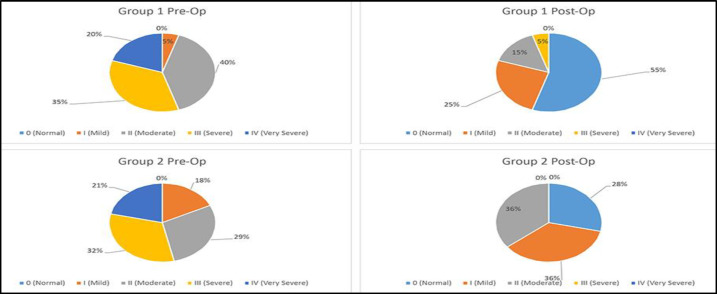
Comparative characteristics of snoring in Groups 1 and 2 pre- and post-operation

- The pre-operation AHI median of 22.1 episodes per hour declined to 14.9 episodes per hour (p<0.05) after surgery: In a similar manner, the AHI varying between 16.6 and 24.9 episodes per hour pre-operatively (2^nd^ degree or moderate sleep apnoea) improved 2 months later after the surgery: 8.3-14.8 episodes per hour (2^nd^ degree or moderate sleep apnoea) were observed in half of the sample postoperatively while 15.1-17.6 episodes per hour (1^st^ degree or mild sleep apnoea) were observed in the other half (p<0.05) (Figure 1).

- Pre-operation median of nasal airflow volume of 167.0 cm^3^ rose to 609.5 cm^3^ post-operatively in a statistically significant manner (p<0.05). While nasal breathing difficulty was deemed moderate in 6 patients (30%), severe in 10 patients (50%) and very severe in 4 patients (25%) pre-operatively, 17 patients (85%) reported full recovery of nasal breathing postoperatively and only 3 patients (15%) reported mild difficulty (p<0.05) (Figure 2).

- A statistically significant improvement was also noticed in snoring. All 4 pre-operative cases of very severe (IV) snoring were no longer severe after the surgery, and 11 patients enjoyed complete elimination of snoring. Positive outcomes were noted in patients with moderate (II) and severe (III) degrees of snoring, with the number of patients with moderate snoring decreased from 8 to 3 and the number of those with severe snoring decreased from 7 to 1 (p<0.05 for all cases) (Figure 3).

- Loudness of snoring decreased from 2.7±0.2 to 0.7±0.2.
- Daytime sleepiness median decreased from 10 to 2 (p<0.05).

*Second group*

The second group comprised 28 patients aged 30 to 62. Mean age was 46.4 and the median age was 46.5, 21 participants (75%) were male and 7 (25%) were female.Table 4 presents the parameter changes in the second group after surgery. It shows that 2 months post-operation the second group did not have a tangible change in BMI, and patients had moderate OSAS, whereas the remaining parameters displayed improvement post-operatively. 

**Table 4:** Parameters of patients suffering from moderate OSAS nasal airflow before and after nasal airway surgery and uvulopalatoplasty

**Variable**	**Pre-Op** **(n=28)**	**Post-Op** **(n=28)**	**p value**
BMI (kg/m^2^), median	28.7 (29.2)	28.6 (29.15)	> 0.25 *
Apnoea-hypopnea index (ep./hour), median	20.7 (20.4)	5.8 (5.3)	< 0.05*
subgroups of apnoea-hypopnea index, absolute number (percentage)			
0^0 ^(normal, < 5 ep./hour)	0 (0.0)	11 (39.3)	< 0.05**
I^0^ (mild, 5-15 ep./hour)	0 (0.0)	17 (60.7)	< 0.05**
II^0 ^(moderate, 15-25 ep./hour)	28 (100.0)	0 (0.0)	< 0.05**
III^0 ^(severe, 25 ep./hour)	0 (0.0)	0 (0.0)	-
Nasal breathing difficulty (cm^3^), median	229.5 (189.5)	524.2 (519.5)	< 0.05*
Subgroups of Nasal breathing difficulty, absolute number (percentage)			
0^0 ^(normal, > 500 cm^3^)	0 (0.0)	23 (82.1)	< 0.05**
I^0^ (mild, 300-500 cm^3^)	6 (21.4)	4 (14.3)	< 0.05**
II^0^ (moderate, 180-300 cm^3^)	10 (35.7)	1 (3.6)	< 0.05**
III^0 ^(severe, 60-180 cm^3^)	8 (28.6)	0 (0.0)	< 0.05**
IV^0 ^(very severe, <60 cm^3^)	4 (14.3)	0 (0.0)	< 0.05**
Snoring, absolute number (percentage)			
No snoring	0 (0.0)	8 (28.6)	< 0.05**
I^0 ^(mild)	5 (17.9)	10 (35.7)	< 0.05**
II^0^ (moderate)	8 (28.6)	10 (35.7)	> 0.09**
III^0^ (severe)	9 (32.1)	0 (0.0)	< 0.05**
IV^0^ (very severe)	6 (21.4)	0 (0.0)	< 0.05**
Epworth sleepiness scale, (median)	12.0 (12)	3.5 (4.0)	< 0.05*

*Note.* *Wilcoxon statistical test, **Pearson statistical test

Specifically, the following results were obtained 2 months after

- The AHI median decreased dramatically from 20.4 episodes per hour to 5.3 episodes per hour (p<0.05). Similarly, if the AHI varied between 15-24.5 episodes per hour pre-operation (2^nd^ degree or moderate sleep apnoea) in all 28 patients, 11 patients (39.3%) had normal indexes and the remaining 17 patients (60.7%) reported an improvement of a mild degree (p<0.05) 2 months post-operation (Figure 1).

- The median of nasal breathing difficulty of airflow volume 189.5 cm^3^ pre-operation grew significantly to 519.5 cm^3^ (p<0.05) post-operation. As many as 23 patients (82%) reported complete restoration of nasal breathing, 4 patients (14%) reported mild levels of breathing difficulty and 1 patient (4%) reported moderate breathing difficulty (Figure 2).
- A statistically significant improvement was also observed in snoring. All 6 pre-operation cases of severe (IV) snoring were no longer severe after the surgery, and 8 patients achieved complete elimination of snoring. Similar positive outcomes have been recorded in patients with severe (III) degree of snoring, as the number of these patients has changed from 9 pre-operation to 0 post-operation(p<0.05) (Fig 3).
- Loudness of snoring improved from 2.6±0.2 pre-operation to 1.1±0.2 post-operation.

Daytime sleepiness median improved from 12 pre-operation to 4 post-operation (p<0.05).

***Intergroup statistical analysis***

The last objective of the study was the comparative evaluation the 2-month post operation outcome between the group of patients who had RVTR of inferior turbinate (and septoplasty in case of deviated nasal septum), and the other group who had RVTR of inferior turbinate, RF UPP and soft palate middle section RVTR as a treatment modality.

It has been shown that:

- Although the AHI median of the groups did not differ much pre-operation (22.1 vs 20.4 episodes per hour, p>0.246), and both groups displayed significant improvement 2 months after the surgery (tables 2 and 3), the 2^nd^ group nevertheless displayed more pronounced improvement in AHI median (14.9 vs 5.3 episodes per hour, p<0.05) (Figure 1).
- Although prior to the surgery the groups did not differ significantly by nasal breathing difficulty median (167.0 cm^3^ vs 189.45 cm^3^, p>0.066), and both groups displayed statistically significant improvements 2 months after the surgery (tables 2 and 3), the 1^st^ group displayed a more pronounced improvement (605.5 cm^3^ vs 519.5 cm^3^, p<0.05) (Figure 2).
- Both groups displayed a statistically significant improvement in snoring (tables 2 and 3). However, not very different in terms of snoring severity pre-operation (p>0.112), the 2^nd^ group demonstrated a much higher level of improvement 2 months after the surgery (p<0.002) (Figure 3).
- The groups were not very different from each other in terms of ESS median pre-operation (10.0 vs 12.0, p<0.066), and although both groups demonstrated statistically significant improvements 2 months after the surgery (tables 2 and 3), the 1^st^ group nevertheless displayed a more pronounced improvement post-operation (2.0 vs 4.0, p<0.05).

## Discussion

It is well known that difficult nasal breathing is one of the major etiological factors for OSAS (5-7). 

At the same time, it is important to take into consideration the fact that nasal breathing accounts for 50% of upper respiratory tract resistance. Nasal obstruction causes inspiratory airflow acceleration, mainly in the nasopharyngeal region, and this contributes to the collapse of soft palate and nasopharyngeal tissue, inducing obstructive apnoea (especially in the supine position while sleeping (14,15). 

Analysis of surgical outcomes of the first group made it evident that nasal airway surgery alone was enough to noticeably reduce the loudness of snoring in patients, as reported by their bed partners. Snoring completely disappeared in 5% of patients and substantially improved in 40% of patients. In the 5 % of participants who were suffering with very severe snoring, the snoring grade improved to severe. In the second group, 64% of the patients’ sleep partners were not bothered by the snoring and the results were deemed as satisfactory. It is worth noting that no patients were left with severe or very severe loudness of snoring after surgery in this group.

Many authors have also shown that nasal airway surgery on its own can significantly improve the severity of snoring (6,16). 

For example, according to the results from the study conducted by Sabbe et al., 61% of patients had satisfactory outcome while Low reported that out of 30 patients (17), 15 experienced complete cure from snoring post operation (17), whereas in study by Ellis et al. 39 (31%) of a total of 126 patients had complete cure and 72 patients (57%) experienced improvement (18). In our study the participants in the first group had a reduction in AHI median from 22.1 to 14.2 episodes per hour although moderate OSAS persisted. In patients who suffer from moderate OSAS, nasal airway surgery alone did not result in cure but helped to decrease the AHI. It also helped to improve the daytime ESS median from 10 to 2.2. 

The AHI varying between 16.1-24.9 episodes per hour improved to 8.3-17.6 episodes per hour in all patients post-operation. It should be noted that in 55% of patients, OSAS severity was reduced to mild and in 45% - to moderate. 

Many authors have also concluded that in patients suffering from OSAS, nasal airway surgery alone reduces the ESS index and improves quality of life but does not do much for a change in the AHI (6,16,17,20-22).

Compared to the first group, the complex surgical procedures performed in the second group in our study resulted in improvement not only in nasal breathing and daytime sleepiness but also substantially in the AHI: this group had a reduction of AHI median from 20.4 to 5.3 episodes per hour. Normal/low levels of AHI were recorded in 15 patients (54%) and mild levels - in 46%. Studies conducted by other authors also attest that RF UPP and nasal airway restoration surgery contribute to improvement of the AHI and other objective parameters considerably (23).For example, in the study by Lim et al. 66% of patients had 50% reduction in AHI after the treatment with the number of episodes less than 20 per hour (23), while Bassiouny et al. demonstrated sufficient improvement of snoring in 75% patients in a sample of study of 20 patients, with AHI improvement in 50% (24). The study of Choi et al. demonstrated improvements in 56.1% of patients in a sample of 49 patients - API decreased by 50% from pre-operation 45.9 ± 23.4 to post-operation 20.9±22.1 episodes per hour (25). According to the study of Verse et al*.*, UPP combined with tonsillectomy demonstrated 59% effectiveness, as opposed to UPP alone which was effective only in 30% of cases. The long-term success rate was 47.6% after 3 to 7 years of follow-up (26).The results of our study demonstrated that, although the effectiveness of nasal airway restoration is higher in patients of the 1^st^ group than that of the 2^nd^ group, RF UPP and soft palate middle section RVTR paired with nasal airway surgery resulted in not only better nasal breathing, but also a more tangible improvement in the indices of AHI, ESS and the alleviation of snoring.

## Conclusion

Based on the outcome of our study, it is possible to state that although nasal airway restoration alone can improve symptoms of daytime sleeping, snoring intensity and the AHI in patients with moderate OSAS, that improvement is not substantial. 

On the other hand, nasal airway surgery combined with UPP is a more efficient method of treating moderate OSAS that significantly improves its symptoms and parameters including snoring intensity, ESS and AHI. We conclude that patients with moderate OSAS who suffer from not only difficult nasal breathing but also have nasopharyngeal luminal narrowing should be advised to have nasal airway surgery combined with UPP for a better outcome.
